# Percentage Body Fat Corresponding to Korean BMI Classification for Obesity in a Nationally Representative Population

**DOI:** 10.3390/nu18121935

**Published:** 2026-06-15

**Authors:** Yong Hee Hong, Hyeoijin Kim, Ji-Hee Kim, Youngjin Jo, Yeseul Lee, Sochung Chung, Chul-Hyun Kim

**Affiliations:** 1 Department of Pediatrics, Soonchunhyang University Bucheon Hospital, Soonchunhyang University College of Medicine, Bucheon 14584, Republic of Korea; hongyonghee@schmc.ac.kr; 2 Department of Physical Education, Korea National University of Education, Chungju 28173, Republic of Korea; aries408@knue.ac.kr; 3 Department of Medical Science, Soonchunhyang University, Asan 31538, Republic of Korea; jhk1111@sch.ac.kr; 4 Institute ofMolecular Metabolism Innovation, Soonchunhyang University, Asan 31538, Republic of Korea; 5 Department of Occupational Therapy, Soonchunhyang University, Asan 31538, Republic of Korea; 6 Department of AI-Integrated Sports and Exercise Medicine, Soonchunhyang University, Asan 31538, Republic of Korea; 7 Department of Sports Medicine, Soonchunhyang University, Asan 31538, Republic of Korea; jo222jo@naver.com (Y.J.); kellyrosa12@sch.ac.kr (Y.L.); 8 Department of Pediatrics, Konkuk University Medical Center, School of Medicine, Konkuk University, Seoul 05030, Republic of Korea; 9 Research Institute for Basic Science, Soonchunhyang University, Asan 31538, Republic of Korea

**Keywords:** body fat percentage, body mass index, obesity, bioelectrical impedance analysis, Korean adults, Korea National Health and Nutrition Examination Survey

## Abstract

**Background/Objectives:** Body mass index (BMI) does not distinguish fat mass from lean mass. Gallagher et al. proposed percentage body fat (%BF) ranges at NIH/WHO BMI cutoffs, but no study has derived %BF ranges for the full set of Asian-specific BMI cutoffs adopted in Korea. This study aimed to develop and externally validate prediction equations for %BF from BMI in Korean adults and to derive %BF ranges corresponding to Korean Society for the Study of Obesity (KSSO) BMI cutoffs. **Methods:** Sex-specific equations were developed using survey-weighted linear regression with the 1/BMI transformation in adults aged ≥ 19 years from KNHANES 2022 (*n* = 4466) using InBody 970 BIA, and externally validated in KNHANES 2023 (*n* = 4778). Predicted %BF values were calculated at each KSSO BMI cutoff (18.5, 23.0, 25.0, 30.0, and 35.0 kg/m^2^) across age-group midpoints. **Results:** Final equations were: Men, %BF = 51.98 − 733.89 × (1/BMI) + 0.058 × age (*R*^2^ = 0.544; SEE = 3.95%); Women, %BF = 64.92 − 731.24 × (1/BMI) (*R*^2^ = 0.638; SEE = 3.66%). Age was not significant in women (*p* = 0.82). External validation showed mean bias of −0.10% and calibration slope of 1.02. The predicted %BF at the pre-obese cutoff (BMI 23.0) was 21.5–24.4% in men and 33.1% in women; at class I obesity (BMI 25.0), 24.1–27.0% and 35.7%, respectively. **Conclusions:** This study provides the first nationally representative %BF ranges for all five KSSO BMI cutoffs. The predicted %BF at BMI 25.0 corresponds to %BF-defined obesity in both sexes, and is consistent with the clinical relevance of the lower obesity cutoff adopted for Asian populations.

## 1. Introduction

Body mass index (BMI) is the most widely used measure for classifying body weight status in clinical practice and epidemiological research. The National Institutes of Health (NIH) and World Health Organization (WHO) define overweight as BMI ≥ 2.5 kg/m^2^ and obesity as BMI ≥ 30 kg/m^2^ [1,2]. Recognizing higher cardiometabolic risk at lower BMI values in Asian populations, the Korean Society for the Study of Obesity (KSSO) has adopted lower cutoffs: pre-obese 23.0–24.9, class I obesity 25.0–29.9, class II obesity 30.0–34.9, and class III obesity ≥ 35.0 kg/m^2^ [3,4]. However, BMI does not distinguish fat mass from lean mass, and individuals with identical BMIs may have markedly different body fat levels depending on sex, age, and ethnicity [5].

In a landmark study, Gallagher et al. [6] addressed this limitation by developing prediction equations linking percentage body fat (%BF) with BMI using dual-energy X-ray absorptiometry (DXA) and the four-compartment (4C) model in 1626 adults from three ethnic groups (white, African American, and Asian), evaluated at three universities in the United Kingdom, the United States, and Japan. They proposed %BF ranges at the NIH/WHO BMI cutoffs of 18.5, 25.0, and 30.0 kg/m^2^. However, their study was based on a convenience sample, did not include Korean participants, and predated the lower Asian BMI cutoffs now used in Korea.

In Korea, BMI-defined obesity (≥25 kg/m^2^) exceeds 38% in adults [7]. Despite the widespread clinical use of the KSSO classification, no corresponding %BF reference values have been established from a nationally representative Korean population. Previous studies derived %BF cutoffs for cardiovascular disease risk [8] and normative fat-free mass index references [9] but were based on single-institution convenience samples using earlier-generation BIA devices. A nationally representative update is needed.

The Korea National Health and Nutrition Examination Survey (KNHANES) has measured body composition using segmental multifrequency BIA (InBody 970) since 2022 (9th Phase), providing a unique opportunity to develop nationally representative %BF reference data. Eight-electrode segmental multifrequency BIA achieves a standard error of 1.9 kg for fat-free mass against the four-compartment model [10,11] and has been endorsed by the Asian Working Group for Sarcopenia as an acceptable method for clinical body composition measurement [12].

This study aimed to develop and externally validate sex-specific prediction equations for %BF from BMI in a nationally representative Korean adult sample, and to derive %BF ranges corresponding to the KSSO BMI guidelines. We followed the analytical approach of Gallagher et al. [6], using the 1/BMI transformation, and extended it to Korean-specific BMI cutoffs. Equations were developed using KNHANES 2022 data and externally validated using KNHANES 2023 data.

## 2. Materials and Methods

### 2.1. Study Design and Data Source

This cross-sectional study used data from KNHANES, 9th Phase, 1st year (2022) and 2nd year (2023), conducted by the Korea Disease Control and Prevention Agency (KDCA). KNHANES is a nationally representative survey conducted under the National Health Promotion Act (Article 16), employing complex, stratified, multistage probability cluster sampling of approximately 192 primary sampling units (PSUs), 4800 households, and 10,000 individuals aged ≥ 1 year annually [7].

Health examinations, including BIA body composition assessment, were performed in mobile examination centers by trained technicians following standardized protocols. Data from 2022 served as the development study and 2023 data as the external validation study. Both survey years used the same BIA device (InBody 970), examination protocols, and eligible age range for BIA measurement (≥10 years), ensuring comparability.

### 2.2. Study Population

**Figure 1:**
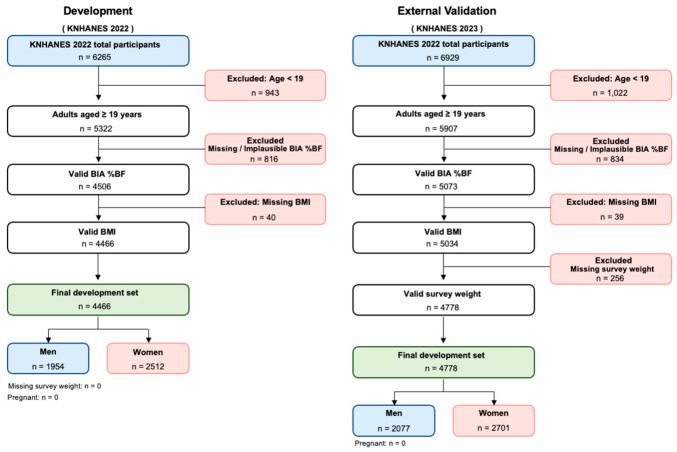
Participant flow diagram. Left panel shows the development set (KNHANES 2022); right panel shows the external validation set (KNHANES 2023). Reasons for exclusion at each step are indicated.

Adults aged ≥ 19 years with valid %BF and BMI data were included. The age threshold of ≥ 19 years corresponds to the statutory definition of adulthood in Korea and to the age range over which the Korean Society for the Study of Obesity (KSSO) applies its adult BMI classification criteria. Individuals aged 18 years and younger are assessed using age- and sex-specific pediatric and adolescent growth references rather than adult BMI cutoffs, and were therefore not included in the present analysis. Participants were excluded if they: (1) were aged <19 years; (2) had missing or implausible BIA %BF (≤0% or ≥80%); (3) had missing BMI; (4) had missing survey weights; or (5) were pregnant. The development set comprised 4466 adults (1954 men, 2512 women), and the validation set 4778 adults (2077 men, 2701 women). The participant flow is presented in Figure 1.

### 2.3. Body Composition and Anthropometric Measurements

Whole-body composition was measured using a segmental multifrequency BIA device (InBody 970; InBody Co., Ltd., Seoul, Republic of Korea) with eight-point tactile electrodes and six frequencies (1, 5, 50, 250, 500, and 1000 kHz). This device directly measures segmental impedance and estimates fat mass, fat-free mass, and segmental lean mass without empirical regression equations. Participants stood barefoot on foot electrodes and gripped hand electrodes after an overnight fast of ≥ 8 h. The primary outcome was whole-body %BF. Eight-electrode segmental multifrequency BIA has been shown to achieve laboratory-grade accuracy comparable to DXA [10,11,12], and our previous cross-validation confirmed high agreement between InBody BIA and DXA (*r* = 0.956–0.960; TE = 2.1–2.3%) for %BF in Korean adults [13].

Standing height was measured to 0.1 cm (Seca 225; Seca GmbH, Hamburg, Germany) and body weight to 0.1 kg (GL-6000-20; G-Tech International Co., Ltd., Uijeongbu, Republic of Korea). BMI was calculated as weight (kg)/height^2^ (m^2^).

### 2.4. BMI Classification

BMI was classified per KSSO 2023 guidelines [3,4]: underweight (<18.5), normal (18.5–22.9), pre-obese (23.0–24.9), class I obesity (25.0–29.9), class II (30.0–34.9), and class III (≥35.0 kg/m^2^). These cutoffs differ from the Western WHO criteria, reflecting higher cardiometabolic risk at lower BMI values in Asian populations [3,6].

### 2.5. Statistical Analysis

All analyses accounted for the KNHANES complex survey design, incorporating stratification, primary sampling units, and appropriate sampling weights. For the development set (2022), the BIA-specific examination weight was used; for the validation set (2023), the general examination weight was applied [14]. Weighted means and standard errors (SEs) were calculated for continuous variables by sex and 10-year age groups. Prediction equations were developed using survey-weighted multiple linear regression following Gallagher et al. [6]. The 1/BMI transformation was used to linearize the curvilinear %BF–BMI relationship [6,15]. Candidate predictors included 1/BMI, sex, age, and their interactions, with forward–backward stepwise selection (entry *p* < 0.05, removal *p* > 0.10). Performance was evaluated using multiple *R*, *R*^2^, and SEE. Residuals were examined for normality (Shapiro–Wilk), homoscedasticity (Breusch–Pagan), and multicollinearity (VIF < 10). Sex-specific equations were developed separately, with internal validation using 10-fold cross-validation. In addition to the combined model, sex-specific prediction equations were developed to allow for potentially different %BF–BMI relationships between men and women. The significance of sex × 1/BMI and sex × age interaction terms in the combined model was assessed to justify sex-stratified analysis. Model adequacy was further evaluated by examining partial regression plots and Cook’s distance for influential observations. The final equations were used to calculate expected %BF at each KSSO BMI cutoff (18.5, 23.0, 25.0, 30.0, 35.0 kg/m^2^) across age-group midpoints (25–75 years), analogous to Gallagher et al. [6]. For comparison, %BF values at the Western WHO cutoffs were also calculated. External validation metrics included: correlation between predicted and observed %BF, mean bias, 95% limits of agreement (Bland–Altman analysis [16]), MAE, RMSE, and calibration slope/intercept. Sensitivity analyses compared nonlinear alternatives (ln[BMI], quadratic, restricted cubic splines) using AIC and cross-validated RMSE. Bias was further examined by age group and BMI category to identify subgroups in which the prediction equations may systematically over- or underestimate %BF. The calibration slope and intercept were estimated by regressing observed %BF on predicted %BF; a slope of 1.0 and intercept of 0 indicate perfect calibration. For comparison, two previously published equations (Gallagher et al. [6] and Sung and Mun [17]) were applied to the validation set, and their *R*^2^, RMSE, mean bias, and calibration slope were evaluated against the present equations. All analyses were performed using Python 3.12 (pandas v2.2, statsmodels v0.14, scipy v1.14, scikit-learn v1.5, matplotlib/seaborn). Two-sided *p* < 0.05 was considered significant. This study followed the STROBE [18] and TRIPOD [19] reporting guidelines. The STROBE checklist is provided in the Supplementary Materials (Table S1).

### 2.6. Use of AI-Assisted Technology

During the preparation of this manuscript, the authors used Claude (Anthropic, Claude Opus 4, San Francisco, CA, USA) to assist with implementing Python code for statistical analyses, organizing manuscript structure, formatting tables and figures, and refining English expression. The authors reviewed and edited all AI-assisted content as needed and take full responsibility for the content of this publication.

## 3. Results

### 3.1. Study Population

Of 6265 participants in KNHANES 2022, 4466 adults (1954 men, 2512 women) met the inclusion criteria (the development study). Of 6929 participants in KNHANES 2023, 4778 adults (2077 men, 2701 women) comprised the validation study (Figure 1). Primary exclusion reasons were age < 19 years (*n* = 943 and 1022, respectively) and missing BIA measurements (*n* = 816 and 834).

### 3.2. Descriptive Characteristics

Table 1 presents weighted descriptive characteristics by sex and age group. In the development set, the weighted mean BMI was 25.0 ± 3.7 kg/m^2^ for men and 23.4 ± 3.8 kg/m^2^ for women. The weighted mean %BF was 24.8 ± 5.8% for men and 32.9 ± 6.1% for women. In women, %BF increased monotonically with age from 31.5% (19–29 years) to 34.9% (≥80 years). In men, %BF showed a U-shaped pattern: relatively stable from age 19–69 (23.9–25.0%) but increasing in older groups (27.0% at ≥80 years), despite declining BMI. The validation set characteristics were closely comparable, supporting the appropriateness of external validation.

By the KSSO classification, 28.8% of men and 44.0% of women had a normal BMI (18.5–22.9 kg/m^2^), whereas 36.2% of men and 22.9% of women were classified as class I obesity (25.0–29.9 kg/m^2^). The weighted mean %BF increased progressively across BMI categories in both sexes.

Values are weighted mean ± SD. *n* = unweighted count. BMI = body mass index; %BF = percentage body fat. Survey-weighted estimates using complex survey design.

### 3.3. Relationship Between %BF and BMI

**Figure 2:**
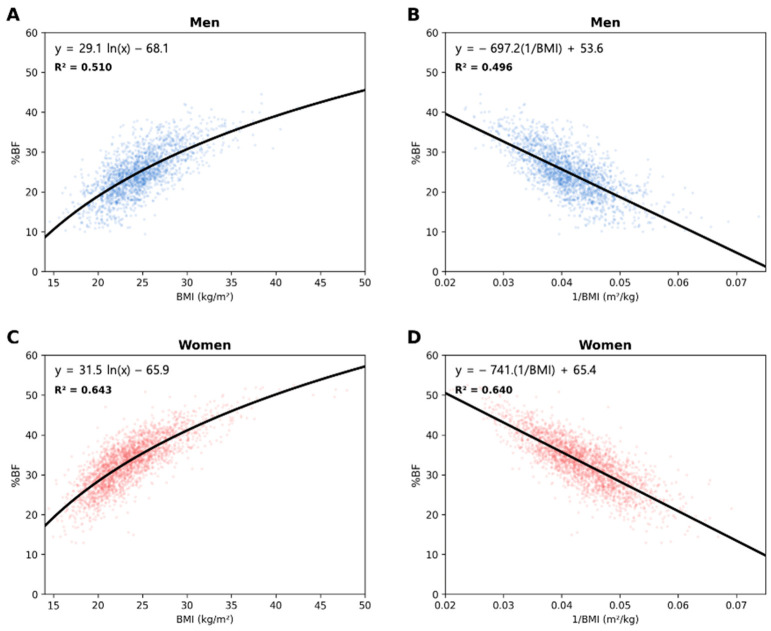
Relationship between percentage body fat (%BF) and body mass index (BMI) in the development set (KNHANES 2022, *n* = 4466). Panels (**A**,**C**) show the curvilinear relationship in men and women, respectively. Panels (**B**,**D**) show the linearized relationship after the 1/BMI transformation. Individual data points are shown as semi-transparent circles; with blue circles representing men (panels (**A**,**B**)) and red circles represeintng women (panels (**C**,**D**)). The solid black line in each panel represents the weighted regression fit.

The %BF–BMI relationship was curvilinear in both sexes (Figure 2A,C). The 1/BMI transformation linearized this association (Figure 2B,D), consistent with Gallagher et al. [6]. The univariate correlation between %BF and 1/BMI was *r* = −0.70 (*R*^2^ = 0.50) in men and *r* = −0.80 (*R*^2^ = 0.64) in women. Alternative transformations yielded comparable multivariate *R*^2^ values (0.723–0.731 for combined-sex models), confirming the appropriateness of the 1/BMI approach.

### 3.4. Prediction Equations

Sex-specific equations were developed using survey-weighted linear regression (Table 2). In men, both 1/BMI and age were significant predictors:

Men: %BF = 51.979 − 733.886 × (1/BMI) + 0.058 × age

(multiple *R* = 0.738, *R*^2^ = 0.544, SEE = 3.95% fat; all *p* < 0.001)

In women, age was not significant (*p* = 0.82), and the final model included 1/BMI only:

Women: %BF = 64.918 − 731.243 × (1/BMI)

(multiple *R* = 0.799, *R*^2^ = 0.638, SEE = 3.66% fat; *p* < 0.001)

The higher intercept in women (64.9 vs. 52.0) indicates approximately 10 percentage points more body fat at the same BMI. The slopes for 1/BMI were similar between sexes (−731 vs. −734). In men, the positive age coefficient (+0.058) indicates %BF increases by approximately 0.6 percentage points per decade at any given BMI, consistent with age-related fat accumulation at stable body weight.

### 3.5. Internal Validation

Ten-fold cross-validation demonstrated stable performance (Table 2). For men, the cross-validated *R*^2^ was 0.532 ± 0.037, RMSE 3.94 ± 0.23%, and mean bias −0.05 ± 0.30%. For women, *R*^2^ was 0.632 ± 0.052, RMSE 3.65 ± 0.15%, and mean bias −0.00 ± 0.24%. Negligible differences between training and cross-validated metrics indicated no overfitting.

### 3.6. %BF Ranges Corresponding to Korean BMI Cutoffs

**Figure 3:**
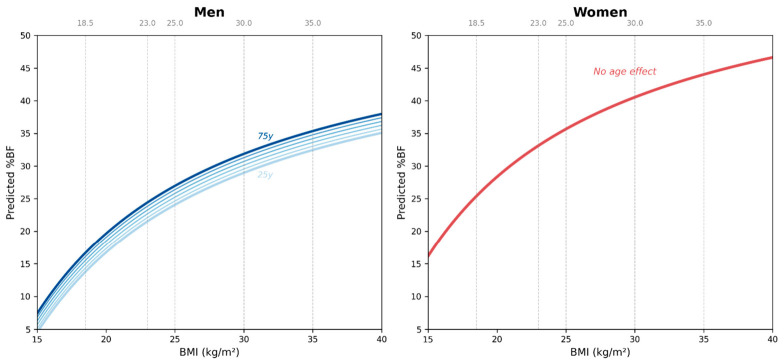
Predicted percentage body fat (%BF) curves across the BMI range for men and women by sex and age with BMI cutoffs. Vertical dashed lines indicate the five KSSO BMI cutoffs (18.5, 23.0, 25.0, 30.0, and 35.0 kg/m^2^). For men, curves are shown at ages 25, 45, and 65 years; the women’s equation does not include an age term. Gray shading represents the 95% prediction interval.

Table 3 presents predicted %BF at each KSSO BMI cutoff for men and women across age-group midpoints (25–75 years). In men, the predicted %BF at the pre-obese cutoff (BMI 23.0) ranged from 21.5% (age 25) to 24.4% (age 75); at class I obesity (BMI 25.0), from 24.1% to 27.0%. In women, the predicted %BF at BMI 23.0 was 33.1% and at BMI 25.0 was 35.7%, with no age variation. At BMI 30.0, predicted values were 29.0–31.9% (men) and 40.5% (women); at BMI 35.0, 32.5–35.4% (men) and 44.0% (women). Figure 3 illustrates the predicted %BF curves with the obesity cutoffs indicated.

For comparison, the predicted %BF at BMI 25.0 and 30.0 in Korean men (aged 30–70) was 1–3 percentage points higher than the corresponding Asian (Japanese) values reported by Gallagher et al. [6]. In Korean women, values were within ±1 percentage point, indicating close agreement despite differences in measurement methods, sample size, and a 25-year interval.

### 3.7. External Validation

**Figure 4:**
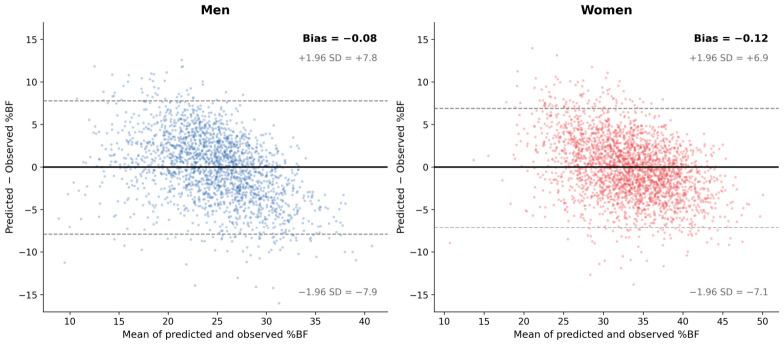
Bland–Altman plots of agreement between predicted and observed %BF in the external validation set (KNHANES 2023) for men (left, blue) and women (right, red). Solid line = mean bias; dashed lines = 95% limits of agreement. Men: bias = −0.08%, LOA = −7.9% to +7.8%. Women: bias = −0.12%, LOA = −7.1% to +6.9%.

When applied to the independent KNHANES 2023 data (Table 4), the prediction equations showed good overall agreement. The Pearson correlation was *r* = 0.862 (combined), mean bias −0.10%, MAE 2.98%, and RMSE 3.77%. The calibration slope was 1.020 and intercept −0.50, indicating near-ideal calibration. The 95% limits of agreement were −7.9% to +7.8% (men) and −7.1% to +6.9% (women) (Figure 4).

Predictive performance across BMI categories is presented in Table S2. Accuracy was highest in the normal-to-class I range (mean bias within ±0.22 percentage points), with systematic underestimation at both BMI extremes (underweight −1.36 and class III obesity −2.39 percentage points), where sample sizes were also smaller.

Age-group-specific calibration statistics are presented in Table S3. Calibration was good across the 30–69-year range (calibration slopes 0.975–1.054; mean bias within ±0.67 percentage points), with a negative mean bias emerging in adults aged ≥ 70 years (−1.29 at 70–79 and −2.03 at ≥80 years), most pronounced in men.

Performance was comparable between sexes: men, *r* = 0.733, bias = −0.08%, RMSE = 4.01%; women, *r* = 0.819, bias = −0.12%, RMSE = 3.58%. Validation *R*^2^ values (men: 0.537; women: 0.671) closely matched development values (men: 0.544; women: 0.638), indicating stable generalization to an independent sample drawn from different primary sampling units and collected in a separate year.

**Figure 5:**
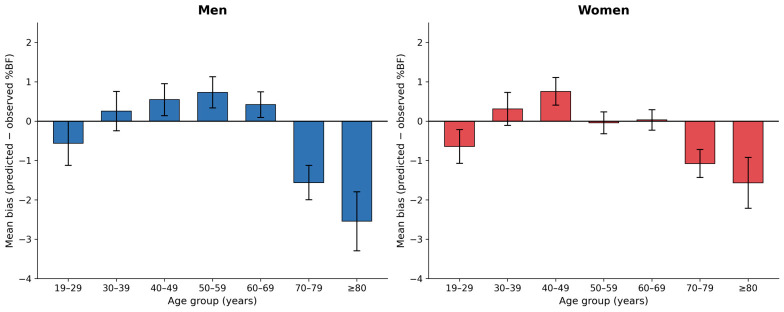
Mean prediction bias by age group in the external validation set (KNHANES 2023) for men (left, blue) and women (right, red). Error bars = 95% CI. Systematic underestimation is observed at ages ≥ 70 years.

Bias was small (+0.3 to +0.8 percentage points) at ages 30–69 years in both sexes (Figure 5).

A systematic negative bias was observed in older adults: −1.6% (men) and −1.1% (women) at age 70–79, and −2.5% (men) and −1.6% (women) at ≥80 years. By BMI category, bias was smallest in the normal and pre-obese ranges and larger at the extremes.

### 3.8. Comparison with Previously Published Equations

To benchmark the present equations against existing models, two previously published equations were applied to the external validation set (KNHANES 2023): the Asian four-compartment equations of Gallagher et al. [6] and the DXA-based Korean equation of Sung and Mun [17]. Predictive performance was evaluated using the same metrics, which were *R*^2^, RMSE, mean bias, and calibration slope, with BIA-derived %BF as the reference outcome (Table 5). The present equations showed the smallest mean bias (−0.10%), the lowest RMSE (3.77%), and the calibration slope closest to unity (1.020). The Gallagher and Sung and Mun equations showed larger mean bias (−1.01% and −1.08%, respectively) and calibration slopes further from unity (0.913 and 0.949). This bias was more pronounced in men (−1.96% for Gallagher and −2.24% for Sung and Mun) than in women (−0.28% and −0.19%). Discrimination was broadly similar across the three equations (*R*^2^ = 0.708 to 0.743).

## 4. Discussion

This study developed and externally validated sex-specific prediction equations for %BF from BMI in a nationally representative Korean adult population and derived %BF ranges corresponding to the KSSO BMI guidelines. The 1/BMI transformation adequately linearized the curvilinear %BF–BMI relationship, consistent with Gallagher et al. [6]. Parsimonious equations achieved moderate-to-good explanatory power (*R*^2^ = 0.54 men, 0.64 women) with SEEs of 3.95% and 3.66%, respectively. The equations demonstrated stable external validation (bias −0.10%; calibration slope 1.02). The predicted %BF at the pre-obese cutoff (BMI 23.0) was 21.5–24.4% in men and 33.1% in women, values not previously available from any nationally representative source.

### 4.1. Comparison with Prior Prediction Equations

Gallagher et al. [6] proposed the foundational 1/BMI approach in 1626 adults from three ethnic groups, achieving *R* = 0.74–0.92 and SEE = 2.8–5.4% across DXA and four-compartment models. Heo et al. [20] replicated this in a nationally representative US sample (NHANES, *n* ≈ 12,900; *R*^2^ = 0.54–0.79) but included no Asian subgroup. More recently, Xu et al. [21] applied machine learning to the same NHANES dataset (*R*^2^ = 0.86) but again without Asian-specific equations. In the Korean context, Sung and Mun [17] developed BMI-based %BF equations using KNHANES 2008–2011 DXA data (*n* ≈ 18,900; *R*^2^ = 0.73). Our study differs in three respects: (1) we used the 1/BMI transformation for methodological continuity with the Gallagher framework, enabling direct cross-study comparison; (2) KNHANES transitioned from DXA to InBody 970 BIA in the 9th Phase, necessitating equations calibrated directly to BIA-measured %BF; and (3) Sung and Mun did not derive %BF ranges at the full KSSO cutoffs, particularly at BMI 23.0, the pre-obese cutoff unique to Asian classifications.

Regarding the second point, the use of BIA-measured %BF as the criterion variable is supported by several considerations. Multi-frequency bioelectrical impedance analysis with segmental eight-electrode technology has demonstrated acceptable agreement with DXA for whole-body fat estimation in healthy adults [10], and is recognized as a validated field method for body composition assessment [11]. In a Korean validation study, BIA showed strong agreement with DXA-measured %BF (*r* = 0.956–0.960; total error = 2.1–2.3%) [13]. Furthermore, the 2025 Lancet Commission on Clinical Obesity recognized BIA as a practical tool for body fat assessment in clinical and epidemiological settings [22]. Because the prediction equations developed in the present study are calibrated directly to BIA-measured %BF without intermediate cross-device conversion, they are immediately applicable to current and future KNHANES cycles that employ the same measurement platform.

Among other Asian populations, Chen et al. [23] found that 82% of Singaporean adults not classified as having obesity by WHO BMI criteria met %BF-defined obesity criteria. Tu et al. [24] established %BF percentile curves for Chinese adults (*n* = 29,064) though without BMI-based prediction equations. These findings, together with ours, underscore that the %BF–BMI relationship in East Asian populations requires ethnicity-specific reference data.

This is borne out by our head-to-head comparison (Table 5): equations derived from DXA or the four-compartment model systematically underestimated BIA-derived %BF, particularly in men, and showed poorer calibration than the present equations, indicating that existing equations do not transfer directly to BIA-derived %BF and supporting the development of equations calibrated to the measurement method used in current Korean practice.

### 4.2. Clinical Implications

The clinical relevance of this work is understood in the context of the growing recognition that BMI-defined and %BF-defined obesity identify overlapping but distinct populations. The 2024 Korea Obesity Fact Sheet [25] reported 41.5% of Korean adults met %BF-defined obesity criteria compared with 38.4% by BMI, illustrating that population-level prevalence estimates can differ depending on the definition used. Yoon et al. [26] similarly demonstrated that BMI alone has limited sensitivity for identifying %BF-defined obesity in Korean adults, highlighting the need for %BF reference values corresponding to each BMI cutoff. Our %BF ranges address this need by providing a quantitative bridge between BMI-based and %BF-based obesity classification. At the KSSO class I obesity cutoff (BMI 25.0), the predicted %BF was 24.1–27.0% in men and 35.7% in women. These predicted values can be interpreted against the outcome-based %BF obesity cutoffs derived by our group using the minimum *p* value approach in 41,088 Korean adults: ≥21% for men and ≥37%BF for women [8]. In men, the predicted %BF at BMI 25.0 exceeds the CVD risk-based cutoff by 3–6 percentage points, indicating that cardiovascular risk is already elevated. In women, the predicted value of 35.7% closely approaches the 37% cutoff, with the 1.3 percentage point difference falling within the standard error of estimate (3.66%). Thus, in both sexes, the KSSO class I obesity cutoff at BMI 25.0 corresponds to %BF-defined obesity, providing body composition-based evidence consistent with the lower obesity cutoff adopted for Asian populations. It should be noted, however, that the outcome-based cutoffs were derived from a single-institution convenience sample more than a decade ago; confirming using nationally representative data with prospective health outcomes would further strengthen this evidence.

Conversely, in women, the %BF at class I obesity (BMI 25.0) was 35.7%, which aligns closely with the commonly used %BF obesity cutoff of 35%. This suggests that the KSSO class I obesity cutoff captures a clinically meaningful level of excess adiposity in women, whereas in men, the pre-obese category alone already signals cardiometabolic risk. These findings have direct implications for clinical practice: health professionals using BMI for obesity screening can now reference the corresponding %BF values to provide more nuanced assessments of body fatness at each BMI cutoff.

The clinical utility of translating BMI cutoffs into corresponding %BF values is threefold. First, the ranges allow clinicians to interpret a patient’s BMI category in terms of expected adiposity, providing an intuitive bridge for practitioners who routinely encounter BMI but rarely %BF. Second, they enable identification of individuals whose measured %BF diverges substantially from the value expected for their BMI such as normal-weight individuals with high %BF, who may be misclassified by BMI alone. Third, they provide a nationally representative reference against which %BF reported by body composition devices can be interpreted. These applications complement, rather than replace, existing BMI-based classification.

### 4.3. Sex Differences

Age was a significant predictor in men (*β* = +0.058/year, *p* < 0.001) but not in women (*p* = 0.82), consistent with Gallagher et al. [6]. In men, lean mass loss proceeds more rapidly in absolute terms with aging, yielding progressive %BF increases not captured by BMI [27]. In women, the age-related %BF increase is more closely paralleled by concurrent BMI changes, particularly around menopause, such that BMI alone captures the aging effect. The intercept difference (64.9 vs. 52.0) reflects the approximately 10-percentage-point biological sex difference in body fat [11].

In women, age did not significantly contribute to the model (*p* = 0.82) and was therefore excluded in accordance with the principle of model parsimony. This likely reflects that the BMI–%BF relationship is stronger in women (*R*^2^ = 0.638) than in men (R^2^ = 0.544), such that BMI alone captures most of the explainable variance in adiposity. Furthermore, the relative stability of body fat percentage across adulthood in women—particularly the attenuation of age-related change after menopause—may reduce the incremental predictive value of age once BMI is accounted for.

### 4.4. Broader Implications

The close agreement between our Korean BIA-derived values and the Japanese 4C-derived values of Gallagher et al. [6], despite a 25-year interval and different methods, suggests stability in the East Asian %BF–BMI relationship. At BMI 25, Korean men had predicted %BF of approximately 25%, compared with 20–22% in white and African American men [20], directly consistent with the rationale for lower BMI cutoffs in Asian populations. This has implications for all East and Southeast Asian countries that have adopted or are considering the WHO Asia-Pacific BMI criteria, as recently endorsed by the Lancet Commission on Clinical Obesity [22].

This stability contrasts with the well-documented ethnic differences between Asian and Western populations: the 3–5 percentage point difference in %BF at the same BMI between East Asian and Western populations provides independent physiological justification for the lower BMI cutoffs adopted in the WHO Asia-Pacific guidelines. Our nationally representative data strengthen the evidence base for these cutoffs by demonstrating the magnitude of the BMI–body fat discordance at each classification level.

Notably, the relationship between BMI and %BF is further modified by age. As shown in our data, older adults exhibited higher %BF at any given BMI than younger adults, reflecting age-related changes in body composition such as the relative loss of lean mass and redistribution of adipose tissue. This age dependency reinforces that a single BMI value may correspond to substantially different levels of adiposity across the lifespan, and it underlies the inclusion of age as a predictor in our male equation.

### 4.5. Considerations Regarding BIA

Unlike Gallagher et al. [6] and Sung and Mun [17], whose equations were derived from four-compartment or DXA measurements, our equations estimate BIA-derived %BF from BMI. Several considerations support this design. Multifrequency BIA is an established field method for body composition assessment [11,12]: it predicts fat-free mass with a standard error of 1.9 kg against the four-compartment model [10], and a Korean validation study reported strong agreement with DXA-measured %BF (*r* = 0.956–0.960; total error 2.1–2.3%) [13], although a systematic offset persists at the individual level. DXA itself is not a criterion standard [6], and the 2025 Lancet Commission on Clinical Obesity endorsed BIA as a practical tool for body fat assessment in clinical and epidemiological settings [22]. Beyond these validity considerations, BIA is well suited to the specific aims of this study. First, the objective is to characterize population-level %BF corresponding to the Korean BMI classification rather than to estimate individual adiposity; for population means, a high-reliability method with a consistent offset is appropriate, and the reference values are internally coherent because they are derived from, and applied with, the same method used in current Korean practice, InBody being the most widely used BIA platform in Korean hospitals and health screening centers. Second, MF-BIA enabled body composition measurement in a large, nationally representative sample at a scale not feasible with the four-compartment model or DXA, and it is the method adopted by KNHANES from its 9th Phase. Third, because KNHANES will continue to use MF-BIA, these reference values establish a methodologically consistent baseline for future secular-trend analyses. We nonetheless emphasize that BIA-derived %BF is not interchangeable with criterion-standard measurements at the individual level.

### 4.6. Strengths and Limitations

Strengths include: (1) first %BF ranges for all five KSSO cutoffs from a nationally representative probability sample; (2) temporal external validation meeting TRIPOD criteria [19]; (3) parsimonious model structure ensuring broad applicability; and (4) direct comparability with the Gallagher framework providing a 25-year benchmark.

The analytic approach, which retains the 1/BMI transformation used by Gallagher et al. [6], enables direct comparison of predicted %BF values between the present Korean data and the original multiethnic reference, providing a unique 25-year longitudinal benchmark for the stability of the %BF–BMI relationship in East Asian populations. Furthermore, the use of two temporally separated KNHANES samples (2022 development, 2023 validation) with entirely separate participants and independent primary sampling units satisfies the TRIPOD criteria for external validation, strengthening the generalizability of the findings.

Limitations include: (1) The sex-specific equations explained 54% (men) and 64% (women) of %BF variance. This is lower than the combined-sex *R*^2^ of 0.73 reported by Sung and Mun [17], but the difference is largely attributable to modeling strategy rather than poorer performance: their model included sex as a predictor, whereas we report sex-specific equations. Because mean %BF differs markedly between sexes, including sex in a pooled model captures this large between-sex variance and inflates *R*^2^; when our data are modeled on the same pooled basis, the combined-sex *R*^2^ is 0.72–0.74, essentially identical to that of Sung and Mun. Substantial within-sex individual-level variability nonetheless remains (approximately 46% in men and 36% in women unexplained), and the derived ranges should therefore be interpreted as population-level expected values rather than individual predictions. (2) Systematic underestimation was observed in adults aged ≥ 70 years (−1.6 to −2.5 percentage points), most pronounced in men (Table S3). This likely reflects age-related sarcopenic changes—the relative loss of fat-free mass and redistribution of adipose tissue—that are not captured by BMI, such that older adults exhibit higher %BF at any given BMI than younger adults. The systematic underestimation in the oldest groups suggests that separate age-specific equations, particularly a dedicated equation for older adults, might improve predictive accuracy in this population. Given that life expectancy continues to rise and the proportion of older adults is increasing rapidly, accurate body composition assessment in this growing demographic is of increasing public health importance. Clinicians applying the present ranges to adults aged ≥ 70 years should be aware that %BF may be underestimated in this group and dedicated %BF reference equations for older adults warrant further study. (3) Because these findings are based on cross-sectional body composition measurements rather than prospective cardiometabolic outcomes, they should be interpreted as consistent with, rather than confirmatory of, the lower obesity cutoffs; prospective studies linking these %BF ranges to incident cardiometabolic disease would be required to establish a causal basis. (4) The present study included only adults aged ≥ 19 years. Because obesity and excess adiposity often begin early in life and are associated with long-term cardiometabolic risk [28], separate analyses in pediatric populations are warranted. In particular, growth and pubertal development may limit the applicability of adult-based prediction models to children and adolescents [29].

## 5. Conclusions

This study provides the first nationally representative %BF ranges corresponding to all five KSSO BMI cutoffs in Korean adults. The prediction equations demonstrated stable external validation and close agreement with prior Asian reference data. The finding that predicted %BF at BMI 25.0 corresponds to %BF-defined obesity in both sexes is consistent with the clinical relevance of the lower obesity cutoff adopted for Asian populations. These results may inform ethnicity-specific approaches to obesity definition, as recommended by the Lancet Commission on Clinical Obesity [22].

## Figures and Tables

**Table 1:** Weighted descriptive characteristics of the development (KNHANES 2022) and external validation (KNHANES 2023) sets by sex and age group.

		Development Set (KNHANES 2022)					External Validation Set (KNHANES 2023)				
Sex	Age Group	*n*	BMI (kg/m^2^)	%BF (%)	Height (cm)	Weight (kg)	*n*	BMI (kg/m^2^)	%BF (%)	Height (cm)	Weight (kg)
Men	19–29	253	25.0 ± 4.6	23.9 ± 6.9	174.7 ± 7.0	76.5 ± 15.8	243	24.8 ± 4.4	23.5 ± 7.3	174.7 ± 6.1	75.9 ± 14.8
	30–39	258	25.7 ± 4.1	24.8 ± 6.2	175.0 ± 5.5	78.7 ± 13.1	249	25.6 ± 3.9	24.4 ± 6.4	175.2 ± 5.5	78.7 ± 13.4
	40–49	309	25.6 ± 3.5	25.0 ± 5.6	173.8 ± 5.8	77.3 ± 11.7	339	25.4 ± 3.6	24.6 ± 5.5	173.9 ± 6.1	77.0 ± 12.4
	50–59	337	25.1 ± 3.2	24.6 ± 5.1	171.1 ± 5.9	73.7 ± 10.7	363	24.9 ± 3.1	24.4 ± 5.3	170.1 ± 5.4	72.2 ± 9.9
	60–69	416	24.2 ± 3.0	24.6 ± 5.2	168.8 ± 5.8	69.0 ± 10.0	472	24.0 ± 3.0	24.3 ± 5.3	168.5 ± 5.6	68.4 ± 9.8
	70–79	295	23.9 ± 2.9	25.9 ± 5.7	166.6 ± 5.2	66.4 ± 9.1	313	23.5 ± 3.0	26.2 ± 5.7	166.0 ± 5.8	64.9 ± 9.2
	≥80	86	23.0 ± 2.4	27.0 ± 5.6	164.6 ± 5.5	62.4 ± 7.5	98	23.2 ± 2.7	27.0 ± 5.3	164.3 ± 6.7	62.7 ± 9.1
	Total	1954	25.0 ± 3.7	24.8 ± 5.8	171.9 ± 6.7	74.0 ± 12.8	2077	24.8 ± 3.6	24.5 ± 6.0	171.6 ± 6.7	73.2 ± 12.8
Women	19–29	296	22.2 ± 4.4	31.5 ± 6.4	162.3 ± 5.4	58.5 ± 12.1	276	22.3 ± 4.2	31.7 ± 6.7	161.8 ± 5.8	58.4 ± 11.7
	30–39	321	22.9 ± 4.4	31.8 ± 6.6	162.1 ± 5.3	60.1 ± 11.4	298	23.3 ± 4.3	32.3 ± 7.1	161.8 ± 5.2	60.9 ± 11.4
	40–49	453	23.3 ± 3.9	32.3 ± 6.1	161.2 ± 5.5	60.5 ± 10.6	455	23.0 ± 4.0	31.4 ± 6.2	160.9 ± 5.5	59.5 ± 10.9
	50–59	457	23.7 ± 3.3	33.2 ± 5.4	158.4 ± 5.0	59.4 ± 8.7	554	23.7 ± 3.6	33.4 ± 5.8	158.0 ± 5.2	59.1 ± 9.4
	60–69	544	24.2 ± 3.4	34.1 ± 5.7	156.0 ± 5.1	58.9 ± 8.8	616	24.2 ± 3.3	34.1 ± 5.5	155.6 ± 5.1	58.5 ± 8.6
	70–79	339	24.3 ± 3.3	34.6 ± 6.1	153.4 ± 5.4	57.3 ± 8.4	393	24.2 ± 3.4	35.1 ± 6.2	152.5 ± 5.0	56.3 ± 8.5
	≥80	102	23.7 ± 2.9	34.9 ± 4.8	149.8 ± 5.3	53.4 ± 7.9	109	23.8 ± 2.9	35.6 ± 5.3	149.6 ± 4.5	53.4 ± 7.3
	Total	2512	23.4 ± 3.8	32.9 ± 6.1	158.8 ± 6.3	59.0 ± 10.1	2701	23.4 ± 3.8	33.0 ± 6.3	158.3 ± 6.4	58.7 ± 10.2

**Table 2:** Prediction equation parameters and internal validation results from the development study.

	Intercept	1/BMI	Age	*R*	*R* ^2^	SEE (%)	CV-RMSE (%)
Men	51.979	−733.8860	0.0580	0.7380	0.5440	3.95	3.94
Women	64.918	−731.2430	—	0.7990	0.6380	3.66	3.65
Men: %BF = 51.979 − 733.886 × (1/BMI) + 0.058 × age							
Women: %BF = 64.918 − 731.243 × (1/BMI)							

All coefficients *p* < 0.001. Age was not significant in women (*p* = 0.82). SEE = standard error of estimate. CV-RMSE = cross-validated root mean square error from 10-fold cross-validation. 1/BMI = inverse of body mass index. Data from KNHANES 2022.

**Table 3:** Predicted percentage body fat (%BF) ranges at KSSO BMI cutoffs by sex and age group.

Sex	Age (y)	BMI 18.5	BMI 23.0	BMI 25.0	BMI 30.0	BMI 35.0
		Underweight	Pre-Obese	Class I	Class II	Class III
Men	25	13.8 (13.2~14.3)	21.5 (21.2~21.8)	24.1 (23.8~24.4)	29.0 (28.6~29.3)	32.5 (32.0~32.9)
	35	14.3 (13.9~14.8)	22.1(21.9~22.3)	24.7 (24.4~24.9)	29.5 (29.2~29.8)	33.0 (32.6~33.5)
	45	14.9 (14.5~15.3)	22.7(22.5~22.9)	25.2(25.0~25.4)	30.1(29.8~30.4)	33.6(33.2~34.0)
	55	15.5(15.1~15.9)	23.3(23.1~23.5)	25.8(25.6~26.0)	30.7(30.4~31.0)	34.2(33.8~34.6)
	65	16.1(15.6~16.5)	23.8(23.6~24.1)	26.4(26.1~26.6)	31.3(30.9~31.6)	34.8(34.3~35.2)
	75	16.7(16.2~17.1)	24.4(24.1~24.7)	27.0(26.6~27.3)	31.9(31.4~32.3)	35.3(34.8~35.9)
Women	25	25.4 (25.1~25.7)	33.1 (33.0~33.3)	35.7 (35.5~35.8)	40.5 (40.3~40.8)	44.0 (43.7~44.4)
	35	25.4 (25.1~25.7)	33.1 (33.0~33.3)	35.7 (35.5~35.8)	40.5 (40.3~40.8)	44.0 (43.7~44.4)
	45	25.4 (25.1~25.7)	33.1 (33.0~33.3)	35.7 (35.5~35.8)	40.5 (40.3~40.8)	44.0 (43.7~44.4)
	55	25.4 (25.1~25.7)	33.1 (33.0~33.3)	35.7 (35.5~35.8)	40.5 (40.3~40.8)	44.0 (43.7~44.4)
	65	25.4 (25.1~25.7)	33.1 (33.0~33.3)	35.7 (35.5~35.8)	40.5 (40.3~40.8)	44.0 (43.7~44.4)
	75	25.4 (25.1~25.7)	33.1 (33.0~33.3)	35.7 (35.5~35.8)	40.5 (40.3~40.8)	44.0 (43.7~44.4)

Values are predicted %BF from sex-specific equations applied at each KSSO BMI cutoff. In women, predicted %BF is identical across ages because age was not a significant predictor. KSSO = Korean Society for the Study of Obesity.

**Table 4:** External validation performance of the sex-specific prediction equations in the validation study.

	*n*	*r*	*R* ^2^	Bias (%BF)	MAE (%BF)	RMSE (%BF)	95% LoA
Overall	4778	0.862	0.743	−0.10	2.98	3.77	−7.5 to +7.3
Men	2077	0.733	0.537	−0.08	3.18	4.01	−7.9 to +7.8
Women	2701	0.819	0.671	−0.12	2.83	3.58	−7.1 to +6.9
		Slope	Intercept				
Overall		1.020	−0.50				
Men		1.009	−0.15				
Women		1.047	−1.44				
Age group	Men (*n*)	Men (bias)	Men (RMSE)	Women (*n*)	Women (bias)	Women (RMSE)	
19–29	243	−0.56	4.50	276	−0.64	3.68	
30–39	249	+0.26	4.01	298	+0.31	3.69	
40–49	339	+0.55	3.85	455	+0.76	3.89	
50–59	363	+0.73	3.91	554	−0.04	3.34	
60–69	472	+0.42	3.63	616	+0.03	3.29	
70–79	313	−1.56	4.23	393	−1.07	3.75	
≥80	98	−2.54	4.54	109	−1.57	3.77	

Bias = predicted − observed. LoAs = limits of agreement. MAE = mean absolute error. RMSE = root mean square error. Calibration: ideal slope = 1.0, ideal intercept = 0. Data from KNHANES 2023.

**Table 5:** Comparative predictive performance of the present equations and previously published equations applied to the external validation (KNHANES 2023, *n* = 4778), with BIA-derived %BF as the reference outcome.

Prediction Equation	Reference Method	*R* ^2 a^	RMSE	Mean Bias ^b^	Calibration Slope
Present Study (Korean, BIA-based)	BIA	0.743	3.77	−0.10	1.020
Gallagher et al. 2000 (Asian, 4C) [6]	4C	0.733	4.02	−1.01	0.913
Sung and Mun 2017 (Korean, DXA) [17]	DXA	0.708	4.17	−1.08	0.949

All equations were applied to the same external validation sample (KNHANES 2023), with multifrequency BIA (InBody 970)-derived %BF as the reference outcome. ^a^ R^2^ is the squared Pearson correlation between predicted and observed %BF (combined sexes). ^b^ Mean bias is the mean of (predicted − observed); negative values indicate underestimation.

## Data Availability

The data analyzed during this study are derived from KNHANES IX (2022–2023), publicly available from the KDCA (https://knhanes.kdca.go.kr/knhanes/eng/main.do; accessed on 18 March 2026). Statistical code is available from the corresponding author upon reasonable request.

## Supplementary Materials

The following supporting information can be downloaded at: https://www.mdpi.com/article/10.3390/nu18121935/s1, Table S1: STROBE Statement—Checklist of items that should be included in reports of cross-sectional studies; Table S2: Predictive performance across BMI categories in the external validation set (KNHANES 2023, *n* = 4778); Table S3: Age-group-specific calibration statistics in the external validation set (KNHANES 2023, *n* = 4778).

## Abbreviations

The following abbreviations are used in this manuscript:

BIA	bioelectrical impedance analysis
BMI	body mass index
BFM	Body fat mass
DXA	dual-energy X-ray absorptiometry
ECW	Extracellular water
FFM	fat-free mass
FFMI	fat-free mass index
FMI	Fat mass index
KDCA	Korea Disease Control and Prevention Agency
KNHANES	Korea National Health and Nutrition Examination Survey
KSSO	Korean Society for the Study of Obesity
PhA	Phase angle
SE	standard error
TBW	Total body water
%BF	Percent body fat

## References

[1] National Institutes of Health (1998). Clinical Guidelines on the Identification, Evaluation, and Treatment of Overweight and Obesity in Adults: The Evidence Report.

[2] World Health Organization (2000). Obesity: Preventing and Managing the Global Epidemic.

[3] World Health Organization, International Association for the Study of Obesity, International Obesity Task Force (2000). The Asia-Pacific Perspective: Redefining Obesity and Its Treatment.

[4] Haam J.-H., Kim B.T., Kim E.M., Kwon H., Kang J.-H., Park J.H., Kim K.-K., Rhee S.Y., Kim Y.-H., Lee K.Y. (2023). Diagnosis of obesity: 2022 update of clinical practice guidelines for obesity by the Korean Society for the Study of Obesity. J. Obes. Metab. Syndr..

[5] Prentice A.M., Jebb S.A. (2001). Beyond body mass index. Obes. Rev..

[6] Gallagher D., Heymsfield S.B., Heo M., Jebb S.A., Murgatroyd P.R., Sakamoto Y. (2000). Healthy percentage body fat ranges: An approach for developing guidelines based on body mass index. Am. J. Clin. Nutr..

[7] Jeong S.M., Jung J.H., Yang Y.S., Yang Y.S., Kim W., Cho I.Y., Lee Y.-B., Park K.-Y., Nam G.E., Han K. (2024). 2023 Obesity Fact Sheet: Prevalence of Obesity and abdominal obesity in adults, adolescents, and children in Korea from 2012 to 2021. J. Obes. Metab. Syndr..

[8] Kim C.H., Park H.S., Park M., Kim H., Kim C. (2011). Optimal cutoffs of percentage body fat for predicting obesity-related cardiovascular disease risk factors in Korean adults. Am. J. Clin. Nutr..

[9] Kim C.-H., Chung S., Kim H., Park J.-H., Park S.-H., Ji J.W., Han S.-W., Lee J.-C., Kim J.H., Park Y.B. (2011). Norm references of fat-free mass index and fat mass index and obesity subtypes in Korean adults aged 18–89 years. Obes. Res. Clin. Pract..

[10] Bosy-Westphal A., Schautz B., Later W., Kehayias J.J., Gallagher D., Müller M.J. (2013). What makes a BIA equation unique? Validity of eight-electrode multifrequency BIA to estimate body composition in a healthy adult population. Eur. J. Clin. Nutr..

[11] Lohman T.G., Milliken L.A. (2020). ACSM’s Body Composition Assessment.

[12] Chen L.K., Woo J., Assantachai P., Auyeung T.-W., Chou M.-Y., Iijima K., Jang H.C., Kang L., Kim M., Kim S. (2020). Asian Working Group for Sarcopenia: 2019 Consensus Update on Sarcopenia Diagnosis and Treatment. J. Am. Med. Dir. Assoc..

[13] Kim H., Kim C.H., Kim D.W., Park M., Park H.S., Min S.-S., Han S.-H., Yee J.-Y., Chung S., Kim C. (2011). External cross-validation of bioelectrical impedance analysis for the assessment of body composition in Korean adults. Nutr. Res. Pract..

[14] Korea Disease Control and Prevention Agency (2024). KNHANES 9th Phase (2022–2024) Raw Data User Guidelines.

[15] Heymsfield S.B., Allison D.B., Wang Z.M., Baumgartner R.N., Ross R., Bray G.A., Bouchard C., James W.P.T. (1998). Evaluation of total and regional body composition. Handbook of Obesity.

[16] Bland J.M., Altman D.G. (1986). Statistical methods for assessing agreement between two methods of clinical measurement. Lancet.

[17] Sung H., Mun J. (2017). Development and cross-validation of equation for estimating percent body fat of Korean adults according to body mass index. J. Obes. Metab. Syndr..

[18] von Elm E., Altman D.G., Egger M., Pocock S.J., Gøtzsche P.C., Vandenbroucke J.P., Initiative S. (2007). The Strengthening the Reporting of Observational Studies in Epidemiology (STROBE) statement: Guidelines for reporting observational studies. Lancet.

[19] Collins G.S., Reitsma J.B., Altman D.G., Moons K.G.M. (2015). Transparent Reporting of a Multivariable Prediction Model for Individual Prognosis or Diagnosis (TRIPOD): The TRIPOD statement. BMJ.

[20] Heo M., Faith M.S., Pietrobelli A., Heymsfield S.B. (2012). Percentage of body fat cutoffs by sex, age, and race-ethnicity in the US adult population from NHANES 1999-2004. Am. J. Clin. Nutr..

[21] Xu S., Xue Y. (2023). Development and validation of a prediction equation for body fat percentage from measured BMI: A supervised machine learning approach. Sci. Rep..

[22] Rubino F., Cummings D.E., Eckel R.H., Cohen R.V., Wilding J.P.H., Brown W.A., Stanford F.C., Batterham R.L., Farooqi I.S., Farpour-Lambert N.J. (2025). Definition and diagnostic criteria of clinical obesity. Lancet Diabetes Endocrinol..

[23] Chen K.K., Wee S.L., Pang B.W.J., Lau L.K., Jabbar K.A., Seah W.T., Ng T.P. (2021). Relationship between BMI with percentage body fat and obesity in Singaporean adults—The Yishun Study. BMC Public Health.

[24] Tu C., Pan Q., Zou J., Liao L., Chen X., Li Y., Pu X., Ding Y., Luo X. (2025). A study on the construction of body fat percentage percentile curve for adults aged 20–79 in China. Front Public Health.

[25] Cho S., Jung J.-H., Nam G.E., Cho I.Y., Park K.-Y., Jeong S.-M., Han K. (2025). 2024 Obesity Fact Sheet in Korea. J. Obes. Metab. Syndr..

[26] Yoon J.L., Cho J.J., Park K.M., Noh H.M., Park Y.S. (2015). Diagnostic performance of body mass index using the Western Pacific Regional Office of World Health Organization reference standards for body fat percentage. J. Korean Med. Sci..

[27] Forbes G.B. (1999). Longitudinal changes in adult fat-free mass: Influence of body weight. Am. J. Clin. Nutr..

[28] Khwanchuea R., Punsawad C. (2026). Correlations among whole-body fat, bone, and biomarkers in boys and girls with obesity: A cross-sectional study. Ann. Pediatr. Endocrinol. Metab..

[29] Chung S. (2017). Growth and puberty in obese children and implications of body composition. J. Obes. Metab. Syndr..

