# Supplementary material for: Percentage Body Fat Corresponding to Korean BMI Classification for Obesity in a Nationally Representative Population
Source: Nutrients. 2026 Jun 15;18(12):1935. doi: 10.3390/nu18121935 (PMC13305686; doi:10.3390/nu18121935)
Supplement: Supplementary file 1 [file nutrients-18-01935-s001.zip › nutrients-4350161-supplementary.pdf]

Additional file 1

Table S1. STROBE Statement—Checklist of items that should be included in reports of cross-sectional studies

Article: Percentage body fat corresponding to Korean BMI classification for obesity in a nationally representative population

Authors: Hong YH, Kim H, Kim J-H, Jo Y, Lee Yeseul, Chung S, Kim C-H

Item No	Section/Topic	Recommendation	Reported on
**Title and abstract**			
1a		Indicate the study's design with a commonly used term in the title or the abstract	Title ("nationally representative"), Abstract ("cross-sectional" in Methods)
1b		Provide in the abstract an informative and balanced summary of what was done and what was found	Abstract: Background, Methods, Results, Conclusions
**Introduction**			
2	**Background/rationale**	Explain the scientific background and rationale for the investigation being reported	Background, paragraphs 1–3
3	**Objectives**	State specific objectives, including any prespecified hypotheses	Background, paragraph 5 ("This study aimed to…")
**Methods**			
4	**Study design**	Present key elements of study design early in the paper	Methods, Study design ("This cross-sectional study…")
5	**Setting**	Describe the setting, locations, and relevant dates, including periods of recruitment, exposure, follow-up, and data collection	Methods, Study design (KNHANES 2022–2023, mobile examination centres, KDCA)
6	**Participants**	(a) Give the eligibility criteria, and the sources and methods of selection of participants	Methods, Study population (age ≥19, exclusion criteria 1–5, Fig. 5)
7	**Variables**	Clearly define all outcomes, exposures, predictors, potential confounders, and effect modifiers. Give diagnostic criteria, if applicable	Methods: Body composition (%BF = outcome), BMI (predictor), age, sex; BMI classification per KSSO 2023
8	**Data sources/measurement**	For each variable of interest, give sources of data and details of methods of assessment (measurement). Describe comparability of assessment methods if there is more than one group	Methods, Body composition (InBody 970 BIA, 8-electrode, 6 frequencies; Seca 225 stadiometer; GL-6000-20 scale)
9	**Bias**	Describe any efforts to address potential sources of bias	Methods, Statistical analysis (survey-weighted regression, 10-fold CV, external validation); Discussion, Strengths and limitations
10	**Study size**	Explain how the study size was arrived at	Methods, Study population (KNHANES nationally representative design: 192 PSUs, ~10,000/year; final n = 4,466 dev, 4,778 val)
11	**Quantitative variables**	Explain how quantitative variables were handled in the analyses. If applicable, describe which groupings were chosen, and why	Methods, Statistical analysis (1/BMI transformation, age as continuous, sex-stratified; sensitivity analyses with ln[BMI], quadratic, RCS)
12	**Statistical methods**	(a) Describe all statistical methods, including those used to control for confounding	Methods, Statistical analysis (survey-weighted linear regression, forward–backward stepwise, Bland–Altman, calibration slope)

		(b) Describe any methods used to examine subgroups and interactions	Methods, Statistical analysis (sex × 1/BMI and sex × age interaction terms; sex-stratified models)
		(c) Explain how missing data were addressed	Methods, Study population (exclusion criteria for missing BIA/BMI/weights; Fig. 1 flow diagram)
		(d) If applicable, describe analytical methods taking account of sampling strategy	Methods, Statistical analysis (kstrata, psu, wt_bia/wt_itvex; survey-weighted regression)
		(e) Describe any sensitivity analyses	Methods, Statistical analysis (nonlinear alternatives: ln[BMI], quadratic, RCS compared by AIC/CV-RMSE)
**Results**			
13	**Participants**	(a) Report the numbers of individuals at each stage of the study—e.g., numbers potentially eligible, examined for eligibility, confirmed eligible, included in the study, completing follow-up, and analysed. Give reasons for non-participation at each stage. Consider use of a flow diagram	Results, Study population; Fig. 1 (participant flow diagram)
14	**Descriptive data**	(b) Give reasons for non-participation at each stage<br>(a) Give characteristics of study participants (e.g., demographic, clinical, social) and information on exposures and potential confounders	Fig. 1 (age <19 excluded n = 943/1,022; missing BIA n = 816/834; missing BMI n = 40/39; missing weights n = 0/256)<br>Results, Descriptive characteristics; Table 1 (weighted mean ± SD by sex and age group: BMI, %BF, height, weight)
		(b) Indicate the number of participants with missing data for each variable of interest	Fig. 5 (numbers at each exclusion stage)
15	**Outcome data**	Report numbers of outcome events or summary measures	Results, Descriptive characteristics (%BF by sex, age group, BMI category in Table 1)
16	**Main results**	(a) Give unadjusted estimates and, if applicable, confounder-adjusted estimates and their precision (e.g., 95% confidence interval). Make clear which confounders were adjusted for and why they were included	Results, Prediction equations (regression coefficients, R^2, SEE in Table 2; external validation r, bias, 95% LOA, RMSE, calibration slope in Table 4)
		(b) Report category boundaries when continuous variables were categorized<br>(c) If relevant, consider translating estimates of relative risk into absolute risk for a meaningful time period	Methods, BMI classification (KSSO cutoffs: 18.5, 23.0, 25.0, 30.0, 35.0); Results, Table 3 (%BF at each cutoff)<br>Not applicable (prediction model, not risk estimation)
17	**Other analyses**	Report other analyses done—e.g., analyses of subgroups and interactions, and sensitivity analyses	Results: Internal validation (10-fold CV); bias by age group and BMI category (Table 4, Fig. 4); Gallagher comparison (Table 5)
**Discussion**			
18	**Key results**	Summarise key results with reference to study objectives	Discussion, paragraph 1
19	**Limitations**	Discuss limitations of the study, taking into account sources of potential bias or imprecision. Discuss both direction and magnitude of any potential bias	Discussion, Strengths and limitations (variance explained 54–64%; age ≥70

			underestimation −1.6 to −2.5%p; cross-sectional design)
20	**Interpretation**	Give a cautious overall interpretation of results considering objectives, limitations, multiplicity of analyses, results from similar studies, and other relevant evidence	Discussion: Comparison with prior equations; Clinical implications; Broader implications; Considerations regarding BIA
21	**Generalisability**	Discuss the generalisability (external validity) of the study results	Discussion, Broader implications (East/Southeast Asian populations using WHO Asia-Pacific criteria); Limitations (BIA-specific equations)
**Other information**			
22	**Funding**	Give the source of funding and the role of the funders for the present study and, if applicable, for the original study on which the present article is based	Declarations, Funding (Soonchunhyang University Research Fund; no role in design/analysis/writing)

Reference: von Elm E, Altman DG, Egger M, et al. The Strengthening the Reporting of Observational Studies in Epidemiology (STROBE) statement: guidelines for reporting observational studies. Lancet. 2007;370(9596):1453–1457.

Table S2. Predictive performance across BMI categories in the external validation set (KNHANES 2023, n = 4,778).

BMI category	n	Mean bias[a] (%p)	MAE (%p)	RMSE (%p)	Calibration slope
Underweight (<18.5)	216	−1.36	3.61	4.50	0.793
Normal (18.5–22.9)	1,796	+0.10	3.02	3.86	0.986
Pre-obese (23.0–24.9)	1,047	+0.22	2.96	3.70	1.089
Class I obesity (25.0–29.9)	1,416	−0.14	2.85	3.58	1.053
Class II obesity (30.0–34.9)	259	−1.21	2.92	3.71	0.984
Class III obesity (≥35.0)	44	−2.39	3.69	4.43	0.729

[a] Mean bias is the mean of (predicted − observed) %BF; negative values indicate underestimation. Prediction accuracy was highest in the normal-to-Class I range, which encompassed the majority of participants. Systematic underestimation was observed at both BMI extremes (underweight and Class III obesity), where sample sizes were also smaller. %BF = percentage body fat; MAE = mean absolute error; RMSE = root mean square error.

Table S3. Age-group-specific calibration statistics in the external validation set (KNHANES 2023, n = 4,778).

Age group (y)	n	Mean bias[a] (%p)	RMSE (%p)	Calibration slope
19–29	519	−0.60	4.08	1.045
30–39	547	+0.29	3.84	1.054
40–49	794	+0.67	3.88	0.975
50–59	917	+0.27	3.58	1.046
60–69	1,088	+0.20	3.44	1.036
70–79	706	−1.29	3.97	0.999
≥80	207	−2.03	4.15	0.946

[a] Mean bias is the mean of (predicted − observed) %BF; negative values indicate underestimation. Calibration was good across the 30–69-year range. Systematic underestimation emerged in adults aged ≥70 years and was most pronounced in men (mean bias −1.56 %p at 70–79 years and −2.54 %p at ≥80 years), consistent with age-related sarcopenic changes in body composition not captured by BMI. %BF = percentage body fat; RMSE = root mean square error.
